# Cornelia De Lange Syndrome and Cochlear Implantation

**Published:** 2018-11

**Authors:** George Psillas, Stefanos Triaridis, Vasiliki Chatzigiannakidou, Jiannis Constantinidis

**Affiliations:** 1 *Department of ENT, AHEPA University Hospital, Aristotle University of Thessaloniki, Thessaloniki, Greece.*

**Keywords:** Cochlear implantation, Cornelia de Lange syndrome, Child, Hearing loss, Preschool

## Abstract

**Introduction:**

Literature regarding the different degrees of hearing loss in patients with Cornelia de Lange syndrome (CDLS) reports that half of the affected patients exhibit severe to profound sensorineural hearing loss. We present the first pre-school child with CDLS who underwent cochlear implantation for congenital profound sensorineural hearing loss.

**Case Report:**

A 3-year-old boy with CDLS underwent unilateral cochlear implantation for bilateral profound sensorineural hearing loss. He had characteristic facial features, bushy eyebrows and synophrys, limb anomalies, growth and mental retardation. Based on the results of postoperative speech perception and production tests, his gain in language skills and expressive vocabulary was modest. However, a cochlear implantation had a significant effect on auditory development, in terms of making him aware of sound localization and the different types of environmental sound.

**Conclusion:**

Criteria for cochlear implantation are expanding and now include children with disabilities in addition to deafness, such as those with CDLS. Profoundly hearing-impaired children affected by borderline mental retardation should be considered as potential candidates for cochlear implantation.

## Introduction

Cornelia de Lange syndrome (CDLS), which is characterized by mental retardation and a characteristic appearance, was first described in 1933 by the Dutch pediatrician Cornelia de Lange. The features of this syndrome include low-set hairline front and back, low-set ears, long eyelashes, bushy eyebrows and synophrys (joined eyebrows). Other clinical symptoms are a short upturned nose, thin lips, a crescent-shaped mouth, low birth weight, developmental delay, microcephaly, excessive body hair, small hands and feet, vision abnormalities, seizures, cleft palate, gastrointestinal and heart disorders. The syndrome occurs in approximately 0.5–1 of every 10,000 live births. Most cases are sporadic and result from autosomal dominant mutation in the NIPBL and SMC3 genes (1).

It was previously suggested that hearing loss could be an associated feature of CDLS (2–7). In this paper, we present the first pre-school child with CDLS who underwent unilateral cochlear implantation for congenital profound sensorineural hearing loss.

## Case Report

A 3-year-old boy of Roma origin, clinically diagnosed with CDLS, was referred to the audiology clinic of our center for hearing impairment. His main clinical characteristics were low birth weight, delayed growth, bushy eyebrows with synophrys and excessive body hair. He did not have cleft palate, midfacial developmental anomalies, upper limb malformations or motor delays. Due to the poor compliance of his parents, he had never undergone any hearing assessment prior to our examination. Informed consent was obtained from the parents to allow their child to participate in the study.

**Fig1:**
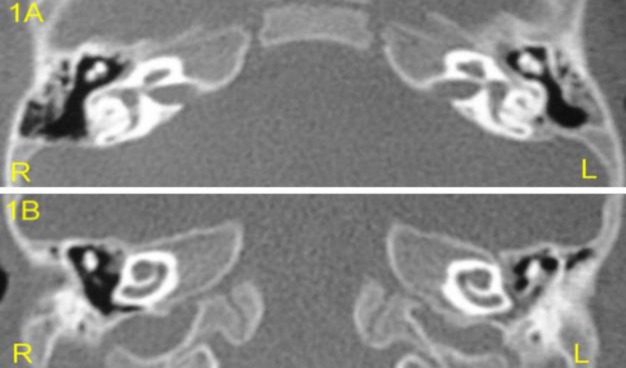
Pre-operative axial (A) and coronal (B) computed tomography scan showing patent basal turn of the cochlea on both sides

The child presented with a total absence of speech and was only articulating some sounds; his non-verbal communication was based mainly on eye contact. Otoscopy and tympanometry were normal. The audiological examination, including auditory brainstem responses and otoacoustic emissions, revealed a bilateral profound sensorineural hearing loss. In visual reinforcement audiometry, performed with warble stimulus in free field, no response was obtained at frequencies of 250–8,000 Hz. The child also underwent a routine evaluation by our cochlear implant team child psychiatrist using psychometric tests (including Denver developmental screening test), and was considered to have border mental retardation. A computed tomography (CT) scan of the temporal bone showed normal patency of the basal turn on both sides (Fig. 1A,B).

**Fig2:**
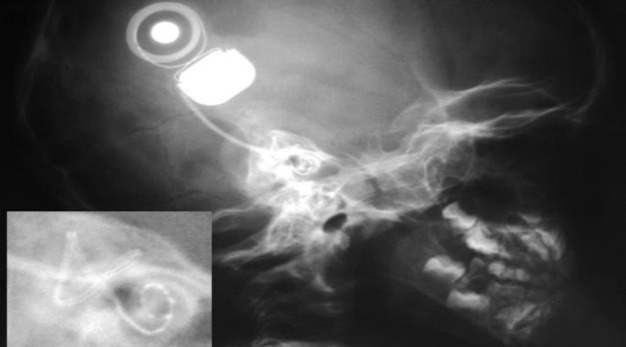
Postoperative X-ray confirming the position of the electrode array into the basal turn of the cochlea (the inset image is a magnification showing the electrode in the basal turn of the cochlea).

Three months after his assessment, the child successfully underwent cochlear implantation of the right ear with Med-el Sonata implant. After typical mastoidectomy and posterior tympanotomy, a Med-el standard electrode was totally inserted via cochleostomy. The postoperative period was uneventful. An X-ray confirmed the intracochlear position of the electrode array (Fig.2). No complications were noted, and the boy was discharged from the hospital on the seventh postoperative day. One month later the child developed bilateral secretory otitis media which was conservatively treated.

In order to measure the child’s auditory-speech perception and speech intelligibility, we used internationally reliable instruments: i) Listening Progress Profile (LiP) to assess his auditory-speech perception; ii) Capacity of Auditory Performance (CAP), which comprised a nonlinear hierarchical scale of auditory receptive abilities, ranging from 0 to 7; and iii) Speech Intelligibility Rating (SIR), a 5-point rating scale that quantifies the speech production abilities of the child (8). Preoperatively, the child's LiP score was very limited at 2%. He could respond to environmental sounds (CAP 1) and his speech was intelligible only when expressed as pre-recognizable words (SIR 1) (Table.1).

**Table 1:** Listening progress profile (LiP). The score (never=0, sometimes=1, always=2) was modified for %.

**Item**	**Pre-op**	**Post-op (2weeks)**	**Post-op (3years)**
Response to environmental sounds	0	0	1
Response to drum (elicited)	0	1	1
Response to musical instrument (elicited)	0	0	1
Response to voice-spontaneous	0	0	1
Response to voice-elicited	0	1	2
Discrimination between two different instruments	0	0	0
Discrimination between loud and quit drum	0	0	0
Discrimination between single and repeated drum	0	0	0
Identification of environmental sounds	0	0	1
Response to oo^a^	0	1	1
Response to ah^a^	0	1	1
Response to ss^a^	0	0	1
Response to mm^a^	0	0	1
Response to ee^a^	0	0	1
Discrimination between long and short speech sounds^a^	0	0	0
Discrimination between single/repeated sounds^a^	0	0	0
Discrimination between loud/quiet speech sounds^a^	0	0	0
Discrimination between two Ling five sounds^a^	0	0	0
Discrimination between all of Ling sounds^a^	0	0	0
Discrimination between two family names of different syllable length^a^	0	0	1
Identification of own name in quiet	1	1	2
Score	1/42, 2%	5/42, 12%	15/42, 36%

a Adapted for the language of our country

**Fig 3:**
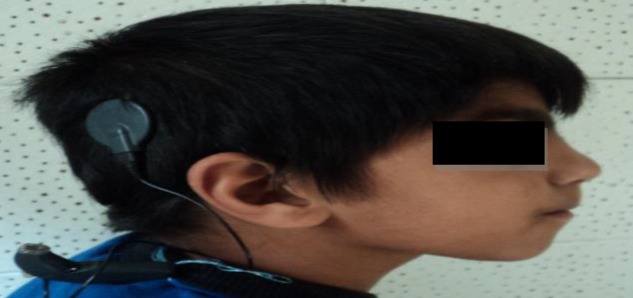
The child with Cornelia de Lange syndrome during follow up examination at six year of age wearing his cochlear implant (with permission)

Two weeks postoperatively, the LiP score of the child had progressed slightly to 12% and his CAP score had increased from 1 to 2 (he could also respond to speech sounds); however, his SIR score remained at 1. At his latest follow up, 3 years postoperatively (at 6 years of age, Fig.3), the child demonstrated a moderate benefit in terms of auditory perception (mean LiP score: 36%), and he managed to identify environmental sounds (CAP 3), although he could not discriminate speech sounds without lipreading (CAP 4). His SIR score was improved from 1 to 2, as his speech was now intelligible when expressed in single words via context and lipreading cues, but it was still unintelligible to a listener who concentrated and lipread (SIR 3). Free-field audiometry using warbled pure tones revealed a satisfactory hearing threshold from 250 to 6,000 Hz in a range from 30 to 45 dB HL. Although the family is bilingual, it was advised to communicate with him only in the language of the home country, in order to match the language of the speech therapy.

## Discussion

The literature regarding hearing loss in patients with CDLS reports that 83% of these cases demonstrate hearing loss of varying degrees (Table.2).

**Table 2:** Rates and degree of hearing loss in CDLS from previous published studies (HL: hearing loss).

**Degree of HL**					
**Author**	**HL (%)**	**Mild**	**Moderate**	**Severe**	**Profound**
Marres^(2)^	3/7 (42)	2	1	-	-
Ichigama^(3)^	2/2 (100)	1	-	-	1
Kaga^(4)^	8/10 (80)	2	-	6	-
Sakai^(5)^	13/13 (100)	-	4	9	-
Egelund^(6)^	2/2 (100)	1	1	-	-
Sataloff^(7)^	38/45 (84)	9	9	-	20
Total	66/79 (83)	15	15	15	21
					

As half of the patients (54%) exhibit severe-to-profound sensorineural hearing loss, cochlear implantation could be considered in this group. The first and only report, by Pulec and Saadat in 1993 (9), of cochlear implantation for congenital deafness due to CDLS concerned a school-aged child (8-year-old girl). They concluded the need to correct hearing loss in children with this syndrome as early as possible. Thus, we report the results of early implantation in a pre-school child (3-year-old boy) with CDLS and bilateral profound sensorineural hearing loss. After his cochlear implantation, the 3-year-old boy showed some benefit in terms of auditory perception, but very limited speech ability. The free-field audiometry hearing threshold ranged from 30 to 45 dB HL; relatively better compared with Pulec and Saadat's case (9) with hearing threshold ranging from 45 to 50 dB HL. However, 3 years after surgery, both cases demonstrated delayed linguistic skills, limited to a few words. There are many reports on the absence of speech in CDLS or the development of minimal vocabulary (10). Children with CDLS are severely handicapped with a combination of mental retardation, physical disability and autism (11). However, the degree of mental retardation in CDLS was found to range from borderline (10%) to mild (8%), moderate (18%), severe (20%) and profound (43%) (11). As in the reported child, a similar case with CDLS and borderline mental retardation was reported to have very delayed expressive language and articulation problems compared with receptive abilities, even with normal hearing (10). It has been shown that patients with CDLS show deficits in language production in comparison with language comprehension and non-verbal cognitive skills (10); in the same study, patients with CDLS and severely delayed language skills or lack of speech had at least one of the five characteristics: moderate-to-severe hearing loss, low birth weight, social retardation, upper limb malformations, and motor delays. Moreover, Sataloff et al. (7) reported that 27 (60%) out of 45 children with CDLS had language limited to a few words.

Nevertheless, cochlear implantation combined with auditory training in this the 3-year-old boy with CDLS had a significant effect on the auditory development in terms of making him aware of sound localization and the different types of environmental sound. We believe that by improving the hearing of a child with this syndrome and multiple congenital deficits, we can alleviate his symptoms and enhance the child’s general function; conversely, untreated deafness can increase disproportionately the symptoms of mental retardation, affecting the development of receptive and expressive skills. The speech perception and production improvement in such children should be carefully monitored over the long term, as improvement can be detected after long-term speech therapy.

Typical temporal bone CT findings have been described in CDLS, such as external auditory canal stenosis, soft tissue opacification of the tympanomastoid cavity, a relatively large malleus head and incus body, hypoplastic cochlea and dilated vestibule (1). In the reported case, no such abnormalities were found (Fig.1A,B) and our Med-el standard electrode of 31-mm in length was completely inserted into the cochleostomy through the posterior tympanotomy.

## Conclusion

The beneficial effect of cochlear implantation on communication in children with additional disabilities, such as in CDLS, is promising. Even though speech perception and production skills remain below average for the child’s calendar age, cochlear implantation can result in improved audiological and quality of life outcomes. The criteria of cochlear implantation are continually expanding to include even individuals with additional disabilities and borderline mental retardation.
